# Network structure underpinning (dys)homeostasis in chronic fatigue syndrome; Preliminary findings

**DOI:** 10.1371/journal.pone.0213724

**Published:** 2019-03-25

**Authors:** James E. Clark, Wan-Fai Ng, Stephen Rushton, Stuart Watson, Julia L. Newton

**Affiliations:** 1 Institute of Neuroscience, Newcastle University, Newcastle, United Kingdom; 2 Institute of Cellular Medicine, Newcastle University, Newcastle, United Kingdom; 3 BCES-Modelling, Newcastle University, Newcastle, United Kingdom; 4 Newcastle upon Tyne Hospitals, NHS Foundation Trust, Newcastle, United Kingdom; James Cook University College of Healthcare Sciences, BRAZIL

## Abstract

**Introduction:**

A large body of evidence has established a pattern of altered functioning in the immune system, autonomic nervous system and hypothalamic pituitary adrenal axis in chronic fatigue syndrome. However, the relationship between components within and between these systems is unclear. In this paper we investigated the underlying network structure of the autonomic system in patients and controls, and a larger network comprising all three systems in patients alone.

**Methods:**

In a sample of patients and controls we took several measures of autonomic nervous system output during 10 minutes of supine rest covering tests of blood pressure variability, heart rate variability and cardiac output. Awakening salivary cortisol was measured on each of two days with participants receiving 0.5mg dexamethasone during the afternoon of the first day. Basal plasma cytokine levels and the *in vitro* cytokine response to dexamethasone were also measured. Symptom outcome measures used were the fatigue impact scale and cognitive failures questionnaire. Mutual information criteria were used to construct networks describing the dependency amongst variables. Data from 42 patients and 9 controls were used in constructing autonomic networks, and 15 patients in constructing the combined network.

**Results:**

The autonomic network in patients showed a more uneven distribution of information, with two distinct modules emerging dominated by systolic blood pressure during active stand and end diastolic volume and stroke volume respectively. The combined network revealed strong links between elements of each of the three regulatory systems, characterised by three higher modules the centres of which were systolic blood pressure during active stand, stroke volume and ejection fraction respectively.

**Conclusions:**

CFS is a complex condition affecting physiological systems. It is important that novel analytical techniques are used to understand the abnormalities that lead to CFS. The underlying network structure of the autonomic system is significantly different to that of controls, with a small number of individual nodes being highly influential. The combined network suggests links across regulatory systems which shows how alterations in single nodes might spread throughout the network to produce alterations in other, even distant, nodes. Replication in a larger cohort is warranted.

## Introduction

Chronic fatigue syndrome (CFS) is a common condition [1], the symptoms of which include unexplained and prolonged fatigue, post-exertional malaise, myalgia, arthralgia, swollen lymph nodes and cognitive impairment [2]. Though historically considered a neuropsychiatric disorder it is now established that pathology extends well beyond this domain [3]. Altered levels of plasma and CSF cytokines have been shown, some elevated and some lowered [4,5], thus the potential for B-cell therapy is being explored with some early promise [6]. Current hypotheses centre on the idea of an altered Th1/Th2 inflammatory profile which possibly results from an overwhelming immunological challenge (e.g. Epstein-Barr virus, cancer, childhood trauma etc.) [7,8]. There is also growing evidence that a combination of autonomic and cardiac dysfunction results in orthostatic hypotension [9], reduced cardiac contractility [10] and impaired muscle recovery after exercise [11], at least in a sub-group of patients [12]. It is unclear whether this represents a central, (probably baroreflex mediated) or a peripheral, cardiovascular effect (or possibly both). More recently, it has also become clear that cortisol levels in patients are frequently lower compared to controls [13], and that this may be consequent on increased negative feedback of the hypothalamic pituitary adrenal (HPA) axis [14].

The findings presented above have yielded several plausible theoretical models [15, 7, 16], though none are yet to emerge as experimentally validated. These models have tended to emphasise the role of allostatic overload as a response to pervasive stress or focus on immune dysregulation as a central cause. However, this ignores the role of signalling within and between networks. For example, pro-inflammatory cytokines (in particular IL-1) invoke increased sympathetic outflow (via indirect bottom-up signalling from the brainstem) which reduces inflammatory cell production at lymphoid tissue and activity by direct cell surface binding. In this way, immune homeostasis is maintained by appropriate sympatho-vagal balance. Similarly, cortisol levels are regulated by circadian CLOCK gene activity in the paraventicular nucleus (PVN) and altered sensitivity of the adrenals to splanchnic sympathetic innervation. Indeed, when challenged these systems work to return to their basal tone through allostasis. It may, therefore, be a category error to focus models on aetiological factors as opposed to the homeostatic alterations which they set in motion. Indeed, while certain events may represent an initial hit, once this has been removed the consequences may well persist in altered systems regulation. In this way, the key pathology which underpins CFS is potentially best understood in computational terms as resulting from altered messages passing amongst homeostatic networks [17]. Indeed, it is a curious observation that much of what has been shown in the literature can be described as a failure of such networks to regulate themselves.

Investigation into this potential phenomenon is impossible under traditional statistical techniques. Instead we require methods capable of evaluating global network structure across a multitude of variables. Theory driven approaches are also difficult in this context, given the vast number of ways in which variables might interact with each other. Mutual information based algorithms have previously been used with much success to investigate cytokine-cytokine and immune-HPA axis network interactions in CFS [18,19] and are well placed to solve these analytic problems. In this paper we use the same approach to compare the network structure of the autonomic nervous system (ANS) in patients and controls, and to describe interactions across the three systems highlighted above in patients only.

## Methods

Favourable ethical opinion was obtained from the Newcastle and North Tyneside Local Research Ethics Committee North East, UK.

### Participants

80 people with CFS, as defined by Fukuda criteria [20], were recruited from a specialist centre in the North East of England as part of a Medical Research Council funded study ‘Understanding the pathogenesis of autonomic dysfunction in chronic fatigue syndrome and its relationship with cognitive impairment’. All CFS subjects were matched to sedentary controls recruited via university volunteer databases, advertisements and “word of mouth”. Participants with co-morbid hypertension or psychiatric illness diagnosed using the SCID-I for research were excluded. Cardioactive medications were withheld for 72 hours prior to assessment.

Controls were recruited through notices in local hospital and university buildings, and healthy relatives of patients attending local support groups were also invited to participate. Identical exclusion criteria to that used in the CFS cohort was used in the recruitment of controls.

Given the aim of the paper was to investigate network structure across a wide range of parameters, only participants in which a complete data set was recorded were included in final analysis. As such, results from 42 patients and 9 controls are reported in the autonomic analysis and 15 patients for the combined network. No controls had complete autonomic, cytokine and HPA axis data and so only a patient combined network is presented.

### Autonomic assessment

Autonomic assessment was conducted at the Clinical Research Facility at the Royal Victoria Infirmary. The Task Force Monitor (TFM, CNSystems, Medizintechnik, Graz, Austria) was used to record and analyse continuous heart rate (electrocardiogram (ECG)),beat-to-beat blood pressure assessment and impedance cardiography. It is reliable and reproducible method of non-invasive autonomic assessment and derives heart rate and blood pressure variability using spectral analysis [21]. Impedance cardiography derives non-invasive cardiac output, stroke volume, ejection fraction and end-diastolic volume [22].

Participants were instructed to eat a light breakfast, avoid caffeine and alcohol on the day of testing and refrain from nicotine for two hours before assessment. All assessments were performed between 9-10am. TFM recordings were taken during a ten minute supine rest a Valsalva manoeuvre [23] and in response to orthostasis (active stand) [24].

### Plasma cortisol measurements

All participants underwent a dexamethasone suppression test [25]. Blood samples were collected into lithium-heparin vacutainers to measure cortisol, at half-hourly intervals from 10am. Five samples were collected on day 1 and 5 on day 2. At 11pm on day 1, participants took dexamethasone by mouth (0.5mg). Within one hour of collection, bloods were spun at 1600g for 10 minutes at room temperature. 2–3 1ml aliquots of plasma were extracted and stored at -80°C until analysis. Plasma cortisol levels were quantified using 15 lot-matched cortisol ELISA kits, supplied by Abcam, which were used according to manufacturer’s protocol. The lower limit of cortisol detection was 2.44ng/ml.

### Serum inflammatory marker measurement

Gel-based specimen tubes were used to collect a day 1 serum sample at 10am for measurement of inflammatory markers. These were spun within 3 hours at 1600g for 10 minutes at room temperature. 2x1ml of serum were extracted and stored at -80°C until analysis. Cytometric bead Array (BD Biosciences) was carried out on serum samples, to measure inflammatory markers, as per the Human Soluble Protein Master Buffer Kit Instruction Manual.

### Symptom assessment outcome measures

All participants completed self-rated phenotype measures assessing functional disease impairment. The Cognitive Failures Questionnaire (CFQ) is a 25 item questionnaire comprised of four domains- memory, names, blunders and distractibility [26]. Participants rate on a five point Likert scale (0 = *“never”*- 4 = *“very often”*) the severity of their everyday cognitive failures. The CFQ possesses good psychometric properties [27]. For the purposes of this study the total questionnaire score was used as a measure of self-rated cognition to provide a more general picture of self-rated impairment across a variety of domains. The Fatigue Impact Scale (FIS) records the impact of fatigue symptoms on daily functioning across cognitive, physical and psychosocial domains [28]. Responses are on a 5 point Likert scale from 0 = *“no problem”* to 4 = *“extreme problem”* and psychometric properties of the FIS are robust [28].

### Analysis

As stated in the introduction, our analytic strategy must be capable of revealing highly interdependent, non-linear relationships between several variables as well as evaluating global network structure. As such, traditional approaches based on shared variance are inappropriate. Instead we used the mutual information (*MI*) based algorithm ARACNE [29], as implemented in the Cytoscape environment [30], to construct networks which describe information passing in each system. Each outcome measure is treated as a node and edges are constructed based on *MI*. Thus a network consists of a collection of variables and the interactions between them. Formally, *MI* between two continuous random variables, X and Y is defined by:

$$MI\left(X;Y\right)=\int \int p\left(x,y\right)ln\frac{p\left(x,y\right)}{p\left(x\right)p\left(y\right)}dxdy$$

We might also note that this is equal to a Kullback-Leibler divergence between the joint and the product of the marginal densities:

MI(X;Y)=KL[p(x,y)||p(x)p(y)]

This means *MI* will be low (near 0) in cases where two variables are statistically independent and high (near 1) when they are likely to occur together. Networks constructed using *MI* therefore describe the statistical dependencies amongst nodes [31].

In order to construct a network, ARACNE creates all possible edges before eliminating edges according to certain criteria [31]. The first is the data processing inequality (DPI) which states that if the link between two nodes is mediated by a third node then:

MI(X;Z)≤min[MI(X;Y),MI(Y;Z)]

[31]

This means that direct links between indirectly interacting nodes will be removed from the final network in favour of higher information yield from indirect paths. The second criterion is an *MI* threshold for edges based on a specified *p*-value threshold. This is calculated by:

$$MI_{0}=\frac{1.062-lnP_{0}}{0.634N+48.7}$$

[29]

This equation is based on empirically derived values as specified in Margolin *et al*. (2006b). In the comparison of ANS networks a *p*-value of 0.01 was used as a modest threshold, though in networks as small as specified here we should not expect a large number of false-positives. In the CFS/ME sample this results in an *MI* threshold of 0.075 and in the control sample a threshold of 0.104. In the combined CFS/ME network, due to the increased number of comparisons a Bonferonni correction was applied resulting in a *p*-value threshold of 0.0002 resulting in an *MI* threshold of 0.16. The performance of the ARACNE algorithm is adequate for data sets containing 100–125 data points [31]. The CFS ANS data contains 462 data points, the controls ANS data 99 data points and the CFS combined network contained 345 data points. Our analysis should therefore be valid and the number of false-positives kept to a minimum.

The modularity of constructed networks was evaluated using the ModuLand family of algorithms [32]. First the influence function for each node(*s*) over each link (*i*,*j*) in the network (*f*_*s*_(*i*,*j*)) s calculated via the LinkLand algorithm [33]. Here a set of nodes, *A*, is iteratively expanded, beginning with the nodes *k* and *l* lying either side of the link (*k*,*l*), until nodes strongly influenced by *k* and *l* are discovered. This is achieved by calculating the set density:

$$d=\frac{\sum_{\left(i,j\right)\in A}w_{i,j}}{\left|A\right|}$$

Here *W*_*i*,*j*_ is the weight of the link (in this case the *MI*) between nodes *i* and *j* and |*A*| is the number of nodes in the set *A* [33]. The density therefore represents the average *MI* of links between the nodes in *A*. The density is then updated by calculating the potential density achieved by adding neighbours of nodes in *A* [33].

If the density of *A* can be increased by doing this then the node which maximises *d’* is added to the set and the processes begins again. This continues in the same fashion until the set density can no longer be increased. The influence function of *s* over elements of *A* is then calculated based on the strength of its links to the other elements and is zero for nodes not in *A* [33]. This is then used to establish a centrality value for each node within a set.

This method will therefore establish a small number of highly influential variables, each in dissociable sets which will form the basis for constructing modules. Indeed, the most influential nodes will form the centre of each module. This centre represents the local maximum of link centrality values and so module number is determined by the number of local maxima within the network. The maxima are then treated as peaks of a hill, where nodes with some influence form the slope and are retained in the module. In determining membership, the Proportional Hill algorithm is used which establishes a set of hill membership values (*H*_*m*_(*i*,*j*)) for each network link (Kovács *et al*., 2010). If the link forms part of the centre of module *K* then its hill membership value is equal to its centrality value and zero for other modules. For all other links, a link will have a high membership value for *K* when neighbouring links also do. If the neighbours of *(i*,*j)* belong to different modules, then their centrality will determine which module *(i*,*j)* becomes a member of. The link membership values are then used to assign membership values to nodes.

Modules are presented graphically by assigning node members a given colour. Given the potentially small number of nodes in modules constructed here, analytic comparison of parameters is not possible.

In evaluating the global structure of networks various parameters for diagnostics are available. Descriptive statistics include number of nodes retained, network diameter (the largest distance between two nodes) and network radius (the maximum length of a shortest path). Statistics used for analytic comparison are described in further detail below.

The statistics used are all measures of the influence of a node over the distribution of information throughout the networks, or the connectivity of a node. In measuring the former we use betweenness (B_c_) of a node, which measures its influence over the interactions between other nodes in a network and stress (S_c_) which describes the number of shortest paths passing through each node. Connectivity was measured using closeness (C_c_), which reflects the speed at which information is distributed through a network by each node, neighbourhood connectivity (N_c_) which is the average connectivity of all the neighbours of a node and topological coefficient (T_c_) which measures the extent to which each node shares neighbours with other nodes in a network. All parameters were calculated using the Network Analyzer plugin [34]in Cytoscape.

Analytic comparison of diagnostic parameters in the CFS/ME and control ANS networks was carried out using independent t-test implemented in the R statistical environment [35]. The same parameters were compared to one another via within-subjects t-test in the combined CFS network.

## Results

### Autonomic function in CFS and healthy controls

**Fig 1:**
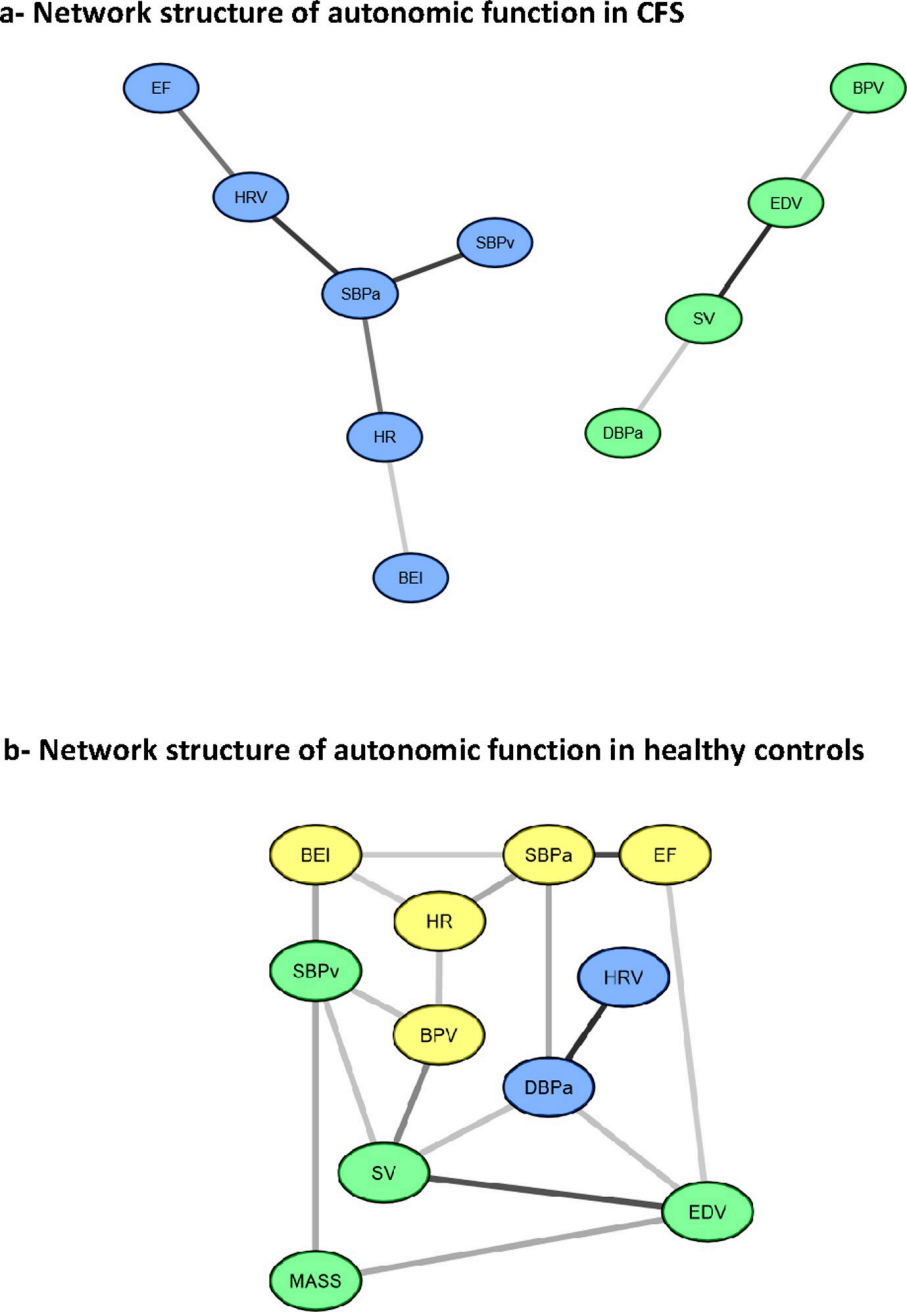
Network structure of autonomic function in a) CFS and b) healthy controls.

The CFS network is shown in Fig 1A. 10 nodes were retained, forming two clearly distinct modules. A large volume of information was directed through mean systolic blood pressure during active stand (C_c_ = 0.71, S_c_ = 16) which also exerted significant influence over other nodes (B_c_ = 0.8). This is further reflected in a lower topological coefficient for this node (T_c_ = 0.33) indicating a central, moderating influence. Within module 2, end diastolic volume (C_c_ = 0.75, S_c_ = 8, B_c_ = 0.67) and stroke volume (C_c_ = 0.75, C_s_ = 8, B_c_ = 0.67) exerted primary control over other nodes. Other nodes in the network were generally less well integrated, and very little information was directed through them (see S1 and S2 Tables for a full list of network parameters).

**Fig 2:**
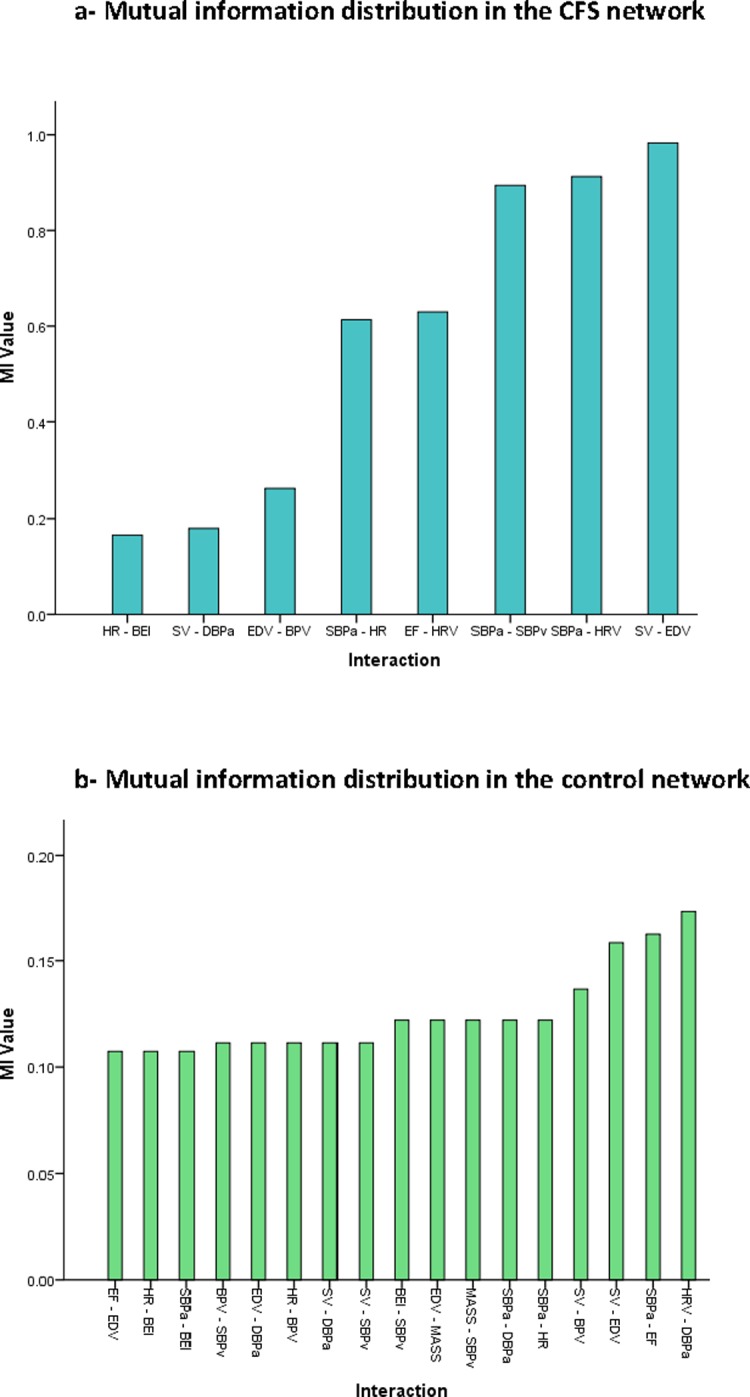
Mutual information distribution in a) the CFS network b) control network. Edge shade is related to MI between nodes. Darker edges indicate a stronger link. Abbreviations: BEI- Baroreflex effectiveness index, BPV- Blood pressure variability, EDV- End diastolic volume, EF- Ejection fraction, HR- Heart rate, HRV- Heart rate variability, MASS- End diastolic wall mass, SBPa- Mean systolic blood pressure during active stand, SBPv- Mean systolic blood pressure during Valsalva, SV- Stroke volume.

The healthy control network is shown in Fig 1B, and has a visibly different structure consisting of 11 total nodes. In this network, three modules were apparent. In contrast to the CFS network, however, there were still significant connections between modules indicating a more evenly distributed structure. This is further supported by a relatively uniform *MI* weighting amongst edges (S3 Table and Fig 2) and by diagnostics (S4 Table) which show no single node was particularly influential or central to the flow of information through the network.

Comparing mean diagnostic values in CFS and controls (Table 1) also suggests a more well connected and evenly distributed network amongst controls. Mean neighbourhood connectivity was significantly higher (*p*<0.0001) as was mean stress (*p* = .01), and there was a trend towards higher mean topological coefficient (*p* = .07). However the average edge *MI* value was significantly higher in the CFS network (*p* = .04).

**Table 1:** Comparison of ANS network diagnostics between CFS and controls.

Interaction	Mutual Information
HRV—DBPa	0.17
MASS—SBPv	0.12
BPV—SBPv	0.11
SV—DBPa	0.11
SV—SBPv	0.11
SV—BPV	0.14
SV—EDV	0.16
EDV—DBPa	0.11
EDV—MASS	0.12
BEI—SBPv	0.12
HR—BEI	0.11
HR—BPV	0.11
EF—EDV	0.11
SBPa—DBPa	0.12
SBPa—BEI	0.11
SBPa—HR	0.12
SBPa—EF	0.16

### The relationship between different regulatory systems in CFS

**Fig 3:**
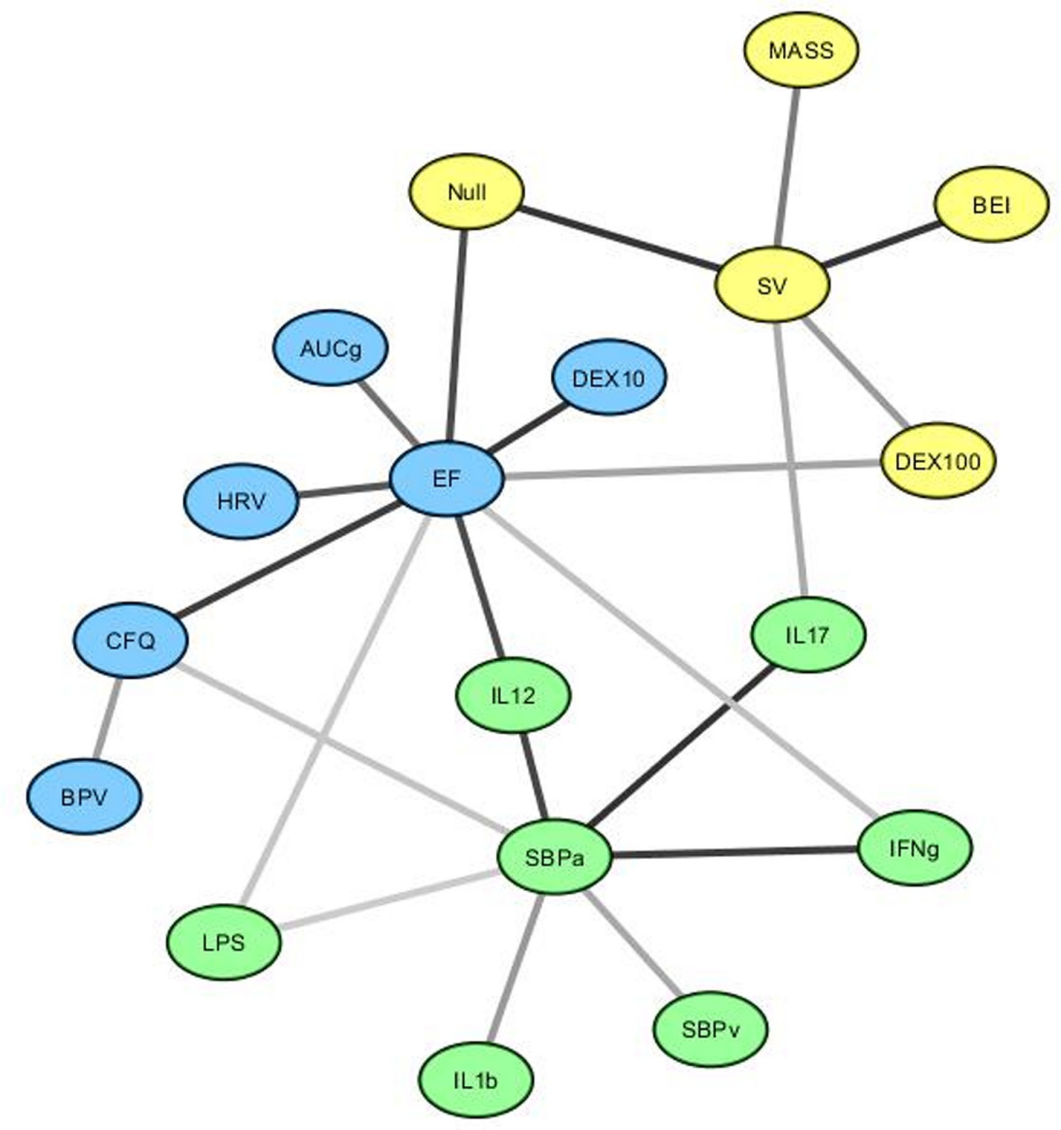
Interactions between the ANS, immune system and HPA axis in CFS. Edge shade is related to MI between nodes. Darker edges indicate a stronger link. Abbreviations: AUCg- Area under the curve with respect to ground, BEI- Baroreflex effectiveness index, BPV- Blood pressure variability, CFQ- Total Cognitive Failures Questionnaire score, DEX10- in vitro glucocorticoid receptor response to 10% dexamethasone solution, DEX100- in vitro glucocorticoid receptor response to 100% dexamethasone solution, EF- Ejection fraction, HRV- Heart rate variability, LPS- in vitro glucocorticoid receptor response to lipopolysaccharide, MASS- End diastolic wall mass, Null, in vitro glucocorticoid receptor response without stimulation, SBPa- Mean systolic blood pressure during active stand, SV- Stroke volume.

**Fig 4:**
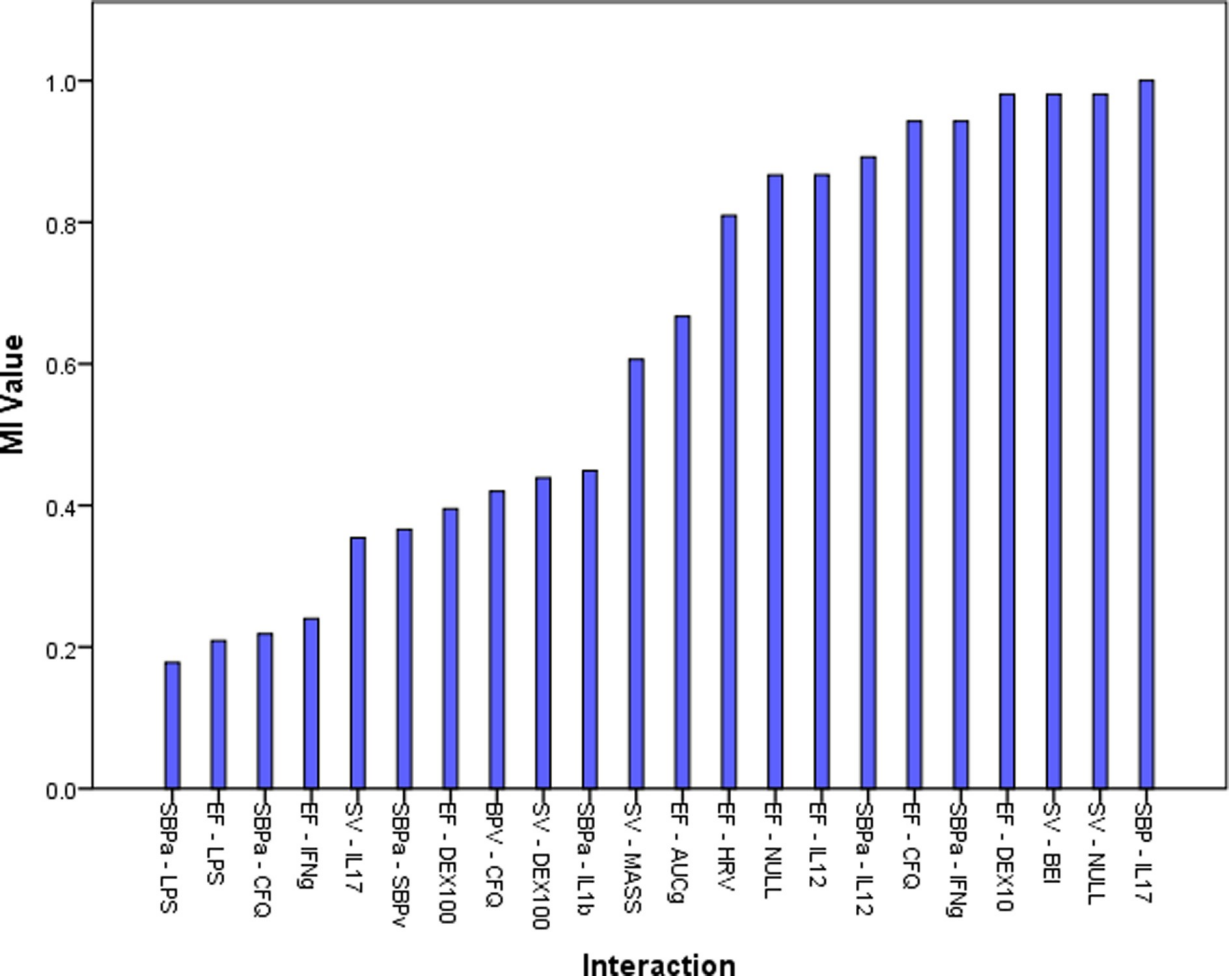
Mutual information distribution for the ANS, immune system, HPA axis network in CFS.

The final network is displayed in Fig 3. 18 nodes were retained which clustered into 3 related modules. The central nodes in these modules were ejection fraction, stroke volume and systolic blood pressure during active stand respectively and these nodes had the three highest betweenness values and were in the four highest nodes for closeness (see S6 Table). The distribution of *MI* values shows a range of link strengths between nodes (Fig 4 and S6 Table) and average link value was high (0.62), though this may be due to the particular influence of the above variables in their respective modules. Mean closeness of each node was significantly higher than betweenness (*p* = .000), and topological coefficient (*p* = .04). Topological coefficient was significantly higher than betweenness (*p* = .02) (Table 2).

**Table 2:** Differences between CFS and control networks.

	CFS Network	Control Network	Mean Difference	P-Value
**Number of Nodes**	10	11	-	-
**Network Diameter**	4	3	-	-
**Network Radius**	2	2	-	-
**Betweenness Centrality**	0.29	0.10	0.19 (0.05, -0.43)	0.10
**Closeness Centrality**	0.56	0.53	0.03 (0.08, -0.13)	0.65
**Neighbourhood Connectivity**	1.97	3.52	-1.55 (-1.93, -1.67)	0.00
**Stress**	4.00	14.54	-10.54 (-17.98, -3.11)	0.01
**Topological Coefficient**	0.23	0.41	-0.18 (-0.38, 0.02)	0.07
**Mean Edge *MI* value**	0.58	0.13	0.45 (0.17, 0.74)	0.01

Brackets indicate 95% confidence interval of the mean difference

## Discussion

Our results show significant differences in the network structure underlying autonomic function in a group of patients with CFS/ME and controls in both general topology and amongst specific nodes and their interactions. Furthermore, in a smaller group of patients the relationships between separate regulatory systems were explored, revealing the network structure underpinning these relationships.

### Autonomic comparison between CFS/ME and controls

In the control group a well distributed, evenly balanced network emerged showing weak to moderate connections amongst modules. This is broadly in keeping with validated, theory-driven computational models [36, 37] and studies on the functional anatomy of the autonomic and cardiovascular systems [38]. This concordance speaks to the validity of our analytic approach and suggests it is capable of establishing underlying network structures in this context, even in a small cohort.

In contrast to the control group, each node in the CFS network was, on average, significantly less well connected to such an extent that one node (end diastolic wall mass) was actually excluded. Despite this, the average edge in the network had a higher *MI* value than in the control network. Furthermore the CFS network was composed of two statistically independent modules the focus of which were three highly influential single nodes (mean systolic blood pressure during active stand, end diastolic volume and stroke volume). This indicates a shift in the quantity of information in the system as well as its distribution, possibly indicating a failure of the system to regulate itself. This view is supported by the loss of direct connections between systolic blood pressure during active stand and baroreflex effectiveness, ejection fraction and end diastolic volume and systolic and diastolic blood pressure during active stand. In the control network these parameters are directly linked. Interestingly, in health these nodes work to maintain adequate perfusion during orthostatic challenge.

Of particular interest is the dissociation between baroreflex and blood pressure, which is crucial in maintaining cerebro-perfusion against gravity. Postural hypotension is frequently reported by patients [39] and a sub-group meet criteria for postural orthostatic tachycardia syndrome (POTS) [12]. Our results might, therefore suggest this is the output of general alterations in autonomic network structure. The visibly stronger link between systolic blood pressure and heart rate in CFS also lends support to this claim- it is possible this is a compensatory effect. The dissociation between systolic and diastolic blood pressure is also intriguing in this regard. We have previously shown both to be significantly reduced at rest in this patient cohort [40] though our results here may suggest different pathological mechanisms. Importantly, this also has implications for targeting of therapeutic intervention- though this can only be fully explored in larger studies.

The dominance of systolic blood pressure during active stand in influencing the CFS network is also in line with previous research highlighting blood pressure abnormalities during orthostatic challenge [41] and may indicate a specific failure to regulate blood pressure is critical to autonomic dysfunction in this patient group. This is in line with observations of heightened sympathetic tone at rest which is reduced during orthostatic challenge [42]. It is possible that when left unchecked by the reflexes which moderate it, failure of blood pressure to respond to orthostatic stress results in a pervasive dysregulation which spreads throughout the ANS, or it may be that some un-regulated external factor inappropriately drives an abnormal blood pressure (e.g. dysregulation on the immune system).

### The relationship between the ANS, immune system and HPA axis in CFS/ME

The broader network examining the interaction between systems showed a wide distribution of *MI* links and the average dependency amongst nodes was high, although this might be the result of a large amount of information flowing through three, well connected nodes. Despite this we found that average closeness was significantly higher than average betweenness and topological coefficient indicating a fast information flow with no node being particularly influential in the network considered as a whole. An explanation for this lies in the fact that these three nodes were highly influential in their respective modules, but with only moderate connections to other nodes. It is important to note that without comparison to a control group we cannot comment on whether this structure is, itself, abnormal. Future work, in a larger group with comparison would shed light on whether topological changes are restricted to single networks (as we have shown in the ANS and has previously been shown in cytokine networks), or whether this also extends to inter-network interaction.

Nevertheless, there are still aspects of the CFS network that warrant consideration. The finding that all the retained cytokines had strong links to systolic blood pressure during active stand is intriguing, particularly given the influential role of this node in the ANS specific network described above. Interactions between the sympathetic system and lymphoid organs are extensive, with reflex anti-inflammatory sympathetic activity occurring after an immune challenge [43]—a phenomenon which is ameliorated via splanchnic lesion in animal models [44]. At the cellular level, Th1 cytokines express β_2_ adrenoceptor to a greater extent than Th2 cells [45] and receptor binding brings about inhibition of IL-12 and IFN-γ [46]. These two cytokines inhibit the production of Th2 cells and so sympathetic activation brings about a relative anti-inflammatory Th2 profile. This process is centrally mediated in a top-down way by the paraventricular nucleus (PVN) [47] forming a classical negative feedback loop. This is in keeping with our results which reflect these dense connections, and show how failure within this loop might spread throughout the broader regulatory network. It is plausible that this might occur either through persistent immune activation which particularly targets the ANS or that there is some failure of the sympathetic system to modulate inflammation properly. Previous findings of elevated pro-inflammatory cytokines [4] and orthostatic hypotension [39] support the former as does our finding that systolic pressure is only linked indirectly to stroke volume and ejection fraction through the effects of IL12 and IL17 respectively. However, the finding that HPA axis nodes are related only to ANS nodes would be expected by a failure of negative feedback at the PVN. Direct comparison of these hypotheses is warranted, in a larger cohort, for conclusions to be drawn.

The place of self-rated cognitive impairment, as measured by CFQ score is also interesting to note. Its only direct links were to three autonomic variables (blood pressure variability, ejection fraction and systolic blood pressure after active stand) which may provide further evidence of a centrally driven autonomic dysregulation- the long term central consequences of which may include cognitive difficulties. A particularly strong link with ejection fraction has been reported in cardiovascular diseases [48], though not in CFS. Ejection fraction is typically preserved in CFS [40] though its place in the autonomic network is different raising the possibility of alterations in processes underlying a normal value. The finding that ejection fraction aligned with a separate module to stroke volume and end diastolic wall mass also lends support to this claim. The direct link with heart rate variability (an indicator of sympathovagal balance) suggests altered sympathetic tone may play a role. Although the relationship between cognitive difficulties and ANS dysregulation is yet to be directly evaluated, there are studies which do provide preliminary evidence for a link. In particular, recent imaging studies have shown altered white matter connectivity in key brain regions [49] and there is evidence for altered brain stem grey matter associated with autonomic dysregulation [50]. The computationally driven network approaches used in this study would prove particularly useful in modelling these relationships.

It is also important to compare our network to the only previous studies using a similar technique to investigate cytokine-cytokine and immune-HPA axis interactions [18,19]. These studies found close relationships between IL12, IFN-γ and IL1β [19] which is in line with our study, though our network may suggest a spurious link via a shared interaction with systolic blood pressure. However, the role of IL17 is quite different, being indirectly linked to these other cytokines in Broderick *et al*. It is unclear why this might be, though its link to the stroke volume centred module may indicate a slightly different role to the other cytokines included here. Further work on this cytokine in CFS may prove fruitful in establishing its exact role. The finding of no direct links between HPA axis and immune variables is quite in contrast to Fuite *et al*. though a mediating role of the ANS in these relationships may offer some explanation. It is also important to acknowledge these differences may be the result of different sample sizes which can only be evaluated via larger cohorts.

## Limitations

The primary limitation of our study is the small sample sizes used. This represents a practical limitation consequent on incomplete data in a large number of study participants. Whilst the validity of the methodology is unaffected by this, it does mean our results cannot be generalised to the wider CFS population. The fact that our results are in line with existing evidence and theoretical models is encouraging but future studies should focus on a more specific set of variables in a large group of patients and controls. Our sample size is also likely to increase the probability of false-negative results, particularly given the quite strict *p*-value threshold applied in the combined network. It is difficult to guard against this without tipping the threshold in favour of false-positives and so, again, larger cohorts are warranted.

It is also important to acknowledge that the particularly small control sample size in the autonomic comparison is likely to impact on our results. Whilst the control network is very much in line with what we would expect, there is still the strong possibility that average MI values taken from a sample of nine may not be representative of the wider population. This increases the likelihood of type 1 errors in our comparison of patient and control autonomic networks.

Again, it is also important to note that the lack of control group in our combined network means we cannot comment on whether the structure is abnormal. Although we can draw conclusions based on comparison with existing data, further work with well-defined, larger sample cohorts is required.

## Conclusions

In this study we examined the underlying network structure of the ANS in CFS and controls. We then examined the relationship between the ANS, immune system and HPA axis in CFS only. Our results showed a fundamental shift in the relationships between components of the ANS, as well as the influence of particular variables, possibly pointing to a failure of the ANS to regulate itself. Furthermore, we found close links between the three regulatory systems in CFS which may show how failures in homeostasis manifest with diffuse changes in a wide variety of outcome measures. Our results raise interesting questions and hypotheses which should be tested in larger cohorts.

## Supporting information

S1 Table Edge parameters in the CFS ANS network. (DOCX)

S2 Table Node parameters in the CFS ANS network. (DOCX)

S3 Table Edge parameters in the control ANS network. (DOCX)

S4 Table Node parameters in the control ANS network. (DOCX)

S5 Table Node parameters in the combined network. (DOCX)

S6 Table Edge parameters in the combined network. (DOCX)

S1 File Variable list–Summary of variables included in the modelling. (DOCX)

S2 File Control autonomics–data related to the controls. (CSV)

S3 File Full network data–data related to the full network. (CSV)

S4 File Patient autonomics–data related to the CFS patients. (CSV)
