# Supplementary material for: Network structure underpinning (dys)homeostasis in chronic fatigue syndrome; Preliminary findings
Source: PLoS One. 2019 Mar 25;14(3):e0213724. doi: 10.1371/journal.pone.0213724 (PMC6433252; doi:10.1371/journal.pone.0213724)
Supplement: S1 Table — (DOCX) [file pone.0213724.s001.docx]

**Supplementary Table 1- Edge parameters in the CFS ANS network**

| Interaction | Mutual Information |
| --- | --- |
| EDV - BPV | 0.26 |
| SV - DBPa | 0.18 |
| SV - EDV | 0.98 |
| EF - HRV | 0.63 |
| HR - BEI | 0.17 |
| SBPa - SBPv | 0.89 |
| SBPa - HRV | 0.91 |
| SBPa - HR | 0.61 |

List of abbreviations:

***HRV***- Heart rate variability, ***SBP_v_***- Mean systolic blood pressure during Valsalva, ***BPV***- Blood pressure variability, ***SV***- Stroke Volume, EDV- End diastolic volume, ***DBP_a_***- Mean diastolic blood pressure during active stand, ***BEI***- Baroreflex effectiveness index, ***HR***- Heart rate, ***EF***- Ejection fraction, ***SBP_a_***- Mean systolic blood pressure during active stand
