# Supplementary material for: Network structure underpinning (dys)homeostasis in chronic fatigue syndrome; Preliminary findings
Source: PLoS One. 2019 Mar 25;14(3):e0213724. doi: 10.1371/journal.pone.0213724 (PMC6433252; doi:10.1371/journal.pone.0213724)
Supplement: S2 Table — (DOCX) [file pone.0213724.s002.docx]

**Supplementary Table 2- Node parameters in the CFS ANS network**

| Node | Betweenness  Centrality | Closeness  Centrality | Neighborhood  Connectivity | Stress | Topological  Coefficient |
| --- | --- | --- | --- | --- | --- |
| BPV | 0.00 | 0.50 | 2.00 | 0 | 0.00 |
| BEI | 0.00 | 0.38 | 2.00 | 0 | 0.00 |
| DBPa | 0.00 | 0.50 | 2.00 | 0 | 0.00 |
| EDV | 0.67 | 0.75 | 1.50 | 4 | 0.50 |
| SV | 0.67 | 0.75 | 1.50 | 4 | 0.50 |
| EF | 0.00 | 0.38 | 2.00 | 0 | 0.00 |
| SBPv | 0.00 | 0.45 | 3.00 | 0 | 0.00 |
| HRV | 0.40 | 0.56 | 2.00 | 8 | 0.50 |
| HR | 0.40 | 0.56 | 2.00 | 8 | 0.50 |
| SBPa | 0.80 | 0.71 | 1.67 | 16 | 0.33 |

List of abbreviations:

***HRV***- Heart rate variability, ***SBP_v_***- Mean systolic blood pressure during Valsalva, ***BPV***- Blood pressure variability, ***SV***- Stroke Volume, EDV- End diastolic volume, ***DBP_a_***- Mean diastolic blood pressure during active stand, ***BEI***- Baroreflex effectiveness index, ***HR***- Heart rate, ***EF***- Ejection fraction, ***SBP_a_***- Mean systolic blood pressure during active stand
