# Supplementary material for: Network structure underpinning (dys)homeostasis in chronic fatigue syndrome; Preliminary findings
Source: PLoS One. 2019 Mar 25;14(3):e0213724. doi: 10.1371/journal.pone.0213724 (PMC6433252; doi:10.1371/journal.pone.0213724)
Supplement: S3 Table — (DOCX) [file pone.0213724.s003.docx]

| Interaction | | Mutual Information | |
| --- | --- | --- | --- |
| HRV - DBPa | | 0.17 | |
| MASS - SBPv | | 0.12 | |
| BPV - SBPv | | 0.11 | |
| SV - DBPa | | 0.11 | |
| SV - SBPv | | 0.11 | |
| SV - BPV | | 0.14 | |
| SV - EDV | | 0.16 | |
| EDV - DBPa | | 0.11 | |
| EDV - MASS | | 0.12 | |
| BEI - SBPv | | 0.12 | |
| HR - BEI | | 0.11 | |
| HR - BPV | | 0.11 | |
| EF - EDV | | 0.11 | |
| SBPa - DBPa | | 0.12 | |
| SBPa - BEI | | 0.11 | |
| SBPa - HR | | 0.12 | |
| SBPa - EF | | 0.16 | |

**Supplementary Table 3- Edge parameters in the control ANS network**

List of abbreviations:

***HRV***- Heart rate variability, ***MASS***- End diastolic wall mass, ***SBP_v_***- Mean systolic blood pressure during Valsalva, ***BPV***- Blood pressure variability, ***SV***- Stroke Volume, EDV- End diastolic volume, ***DBP_a_***- Mean diastolic blood pressure during active stand, ***BEI***- Baroreflex effectiveness index, ***HR***- Heart rate, ***EF***- Ejection fraction, ***SBP_a_***- Mean systolic blood pressure during active stand
