# Supplementary material for: Network structure underpinning (dys)homeostasis in chronic fatigue syndrome; Preliminary findings
Source: PLoS One. 2019 Mar 25;14(3):e0213724. doi: 10.1371/journal.pone.0213724 (PMC6433252; doi:10.1371/journal.pone.0213724)
Supplement: S4 Table — (DOCX) [file pone.0213724.s004.docx]

| **Supplementary Table 4- Node parameters in the control ANS network**   \| Node \| Betweenness Centrality \| Closeness Centrality \| Neighbourhood Connectivity \| Stress \| Topological Coefficient \| \| --- \| --- \| --- \| --- \| --- \| --- \| \| HRV \| 0.00 \| 0.40 \| 4.00 \| 0 \| 0.00 \| \| MASS \| 0.02 \| 0.48 \| 4.00 \| 6 \| 0.6 \| \| SBPv \| 0.13 \| 0.56 \| 3.00 \| 20 \| 0.42 \| \| BPV \| 0.05 \| 0.53 \| 3.67 \| 8 \| 0.48 \| \| SV \| 0.15 \| 0.63 \| 3.75 \| 20 \| 0.38 \| \| EDV \| 0.16 \| 0.56 \| 3.00 \| 24 \| 0.42 \| \| DBPa \| 0.25 \| 0.63 \| 3.25 \| 30 \| 0.34 \| \| BEI \| 0.06 \| 0.53 \| 3.67 \| 10 \| 0.48 \| \| HR \| 0.04 \| 0.50 \| 3.339 \| 6 \| 0.50 \| \| EF \| 0.03 \| 0.48 \| 4.00 \| 8 \| 0.60 \| \| SBPa \| 0.20 \| 0.59 \| 3.00 \| 28 \| 0.36 \|   List of abbreviations:  ***HRV***- Heart rate variability, ***MASS***- End diastolic wall mass, ***SBP_v_***- Mean systolic blood pressure during Valsalva, ***BPV***- Blood pressure variability, ***SV***- Stroke Volume, EDV- End diastolic volume, ***DBP_a_***- Mean diastolic blood pressure during active stand, ***BEI***- Baroreflex effectiveness index, ***HR***- Heart rate, ***EF***- Ejection fraction, ***SBP_a_***- Mean systolic blood pressure during active stand | |  | |
| --- | --- | --- | --- | --- | --- | --- | --- | --- | --- | --- | --- | --- | --- | --- | --- | --- | --- | --- | --- | --- | --- | --- | --- | --- | --- | --- | --- | --- | --- | --- | --- | --- | --- | --- | --- | --- | --- | --- | --- | --- | --- | --- | --- | --- | --- | --- | --- | --- | --- | --- | --- | --- | --- | --- | --- | --- | --- | --- | --- | --- | --- | --- | --- | --- | --- | --- | --- | --- | --- | --- | --- | --- | --- | --- | --- |
|  | |  | |
|  | |  | |
|  | |  | |
|  | |  | |
|  | |  | |
|  | |  | |
|  | |  | |
|  | |  | |
|  | |  | |
|  | |  | |
|  | |  | |
|  | |  | |
|  | |  | |
|  | |  | |
|  | |  | |
|  | |  | |
|  | |  | |
