# Supplementary material for: Network structure underpinning (dys)homeostasis in chronic fatigue syndrome; Preliminary findings
Source: PLoS One. 2019 Mar 25;14(3):e0213724. doi: 10.1371/journal.pone.0213724 (PMC6433252; doi:10.1371/journal.pone.0213724)
Supplement: S5 Table — (DOCX) [file pone.0213724.s005.docx]

| Supplementary table 5   \| Node \| Betweenness  Centrality \| Closeness  Centrality \| Neighborhood  Connectivity \| Stress \| Topological  Coefficient \| \| --- \| --- \| --- \| --- \| --- \| --- \| \| BPV \| 0.00 \| 0.33 \| 3.00 \| 0 \| 0.00 \| \| BEI \| 0.00 \| 0.30 \| 5.00 \| 0 \| 0.00 \| \| MASS \| 0.00 \| 0.30 \| 5.00 \| 0 \| 0.00 \| \| SV \| 0.26 \| 0.43 \| 1.60 \| 152 \| 0.30 \| \| AUCg \| 0.00 \| 0.37 \| 9.00 \| 0 \| 0.00 \| \| DEX100 \| 0.09 \| 0.45 \| 7.00 \| 62 \| 0.55 \| \| DEX10 \| 0.00 \| 0.37 \| 9.00 \| 0 \| 0.00 \| \| Null \| 0.09 \| 0.45 \| 7.00 \| 62 \| 0.55 \| \| HRV \| 0.00 \| 0.37 \| 9.00 \| 0 \| 0.00 \| \| EF \| 0.55 \| 0.57 \| 1.78 \| 340 \| 0.26 \| \| CFQ \| 0.15 \| 0.47 \| 5.67 \| 94 \| 0.42 \| \| LPS \| 0.04 \| 0.45 \| 8.00 \| 44 \| 0.64 \| \| IL12 \| 0.04 \| 0.45 \| 8.00 \| 44 \| 0.64 \| \| IL1b \| 0.00 \| 0.34 \| 7.00 \| 0 \| 0.00 \| \| IL17 \| 0.11 \| 0.43 \| 6.00 \| 60 \| 0.50 \| \| IFNg \| 0.04 \| 0.45 \| 8.00 \| 44 \| 0.64 \| \| SBPv \| 0.00 \| 0.34 \| 7.00 \| 0 \| 0.00 \| \| SBPa \| 0.35 \| 0.50 \| 1.86 \| 232 \| 0.29 \| | |  | |
| --- | --- | --- | --- | --- | --- | --- | --- | --- | --- | --- | --- | --- | --- | --- | --- | --- | --- | --- | --- | --- | --- | --- | --- | --- | --- | --- | --- | --- | --- | --- | --- | --- | --- | --- | --- | --- | --- | --- | --- | --- | --- | --- | --- | --- | --- | --- | --- | --- | --- | --- | --- | --- | --- | --- | --- | --- | --- | --- | --- | --- | --- | --- | --- | --- | --- | --- | --- | --- | --- | --- | --- | --- | --- | --- | --- | --- | --- | --- | --- | --- | --- | --- | --- | --- | --- | --- | --- | --- | --- | --- | --- | --- | --- | --- | --- | --- | --- | --- | --- | --- | --- | --- | --- | --- | --- | --- | --- | --- | --- | --- | --- | --- | --- | --- | --- | --- | --- |
|  | |  | |
|  | |  | |
|  | |  | |
|  | |  | |
|  | |  | |
|  | |  | |
|  | |  | |
|  | |  | |
|  | |  | |
|  | |  | |
|  | |  | |
|  | |  | |
|  | |  | |
|  | |  | |
|  | |  | |
|  | |  | |
|  | |  | |
