# Supplementary material for: Network structure underpinning (dys)homeostasis in chronic fatigue syndrome; Preliminary findings
Source: PLoS One. 2019 Mar 25;14(3):e0213724. doi: 10.1371/journal.pone.0213724 (PMC6433252; doi:10.1371/journal.pone.0213724)
Supplement: S6 Table — (DOCX) [file pone.0213724.s006.docx]

| List of abbreviations:  *AUC_g_*- Area under the curve with respect to ground, *BEI*- Baroreflex effectiveness index, *BPV*- Blood pressure variability, *CFQ*- Total Cognitive Failures Questionnaire score, *DEX10*- *in vitro* glucocorticoid receptor response to 10% dexamethasone solution, *DEX100*- *in vitro* glucocorticoid receptor response to 100% dexamethasone solution, *EF*- Ejection fraction, *HRV*- Heart rate variability, *LPS*- *in vitro* glucocorticoid receptor response to lipopolysaccharide, *MASS*- End diastolic wall mass, *Null*, *in vitro* glucocorticoid receptor response without stimulation, *SBP_a_*- Mean systolic blood pressure during active stand, *SV*- Stroke volume   \| Interaction \| Mutual Information \| \| --- \| --- \| \| BPV - CFQ \| 0.42 \| \| SV - DEX100 \| 0.44 \| \| SV - Null \| 0.98 \| \| SV - IL17 \| 0.35 \| \| SV - BEI \| 0.98 \| \| SV - MASS \| 0.61 \| \| EF - CFQ \| 0.94 \| \| EF - AUCg \| 0.67 \| \| EF - DEX100 \| 0.40 \| \| EF - DEX10 \| 0.98 \| \| EF - LPS \| 0.21 \| \| EF - Null \| 0.87 \| \| EF - IL12 \| 0.87 \| \| EF - IFNg \| 0.24 \| \| EF - HRV \| 0.81 \| \| SBP - CFQ \| 0.21 \| \| SBP - LPS \| 0.18 \| \| SBP - IL12 \| 0.89 \| \| SBP - IL1b \| 0.45 \| \| SBP - IL17 \| 1.00 \| \| SBP - IFNg \| 0.94 \| \| SBP - SBPv \| 0.37 \|   Supplementary Table 6- Edge parameters in the combined network | |  | |
| --- | --- | --- | --- | --- | --- | --- | --- | --- | --- | --- | --- | --- | --- | --- | --- | --- | --- | --- | --- | --- | --- | --- | --- | --- | --- | --- | --- | --- | --- | --- | --- | --- | --- | --- | --- | --- | --- | --- | --- | --- | --- | --- | --- | --- | --- | --- | --- | --- | --- |
|  | |  | |
|  | |  | |
|  | |  | |
|  | |  | |
|  | |  | |
|  | |  | |
|  | |  | |
|  | |  | |
|  | |  | |
|  | |  | |
|  | |  | |
|  | |  | |
|  | |  | |
|  | |  | |
|  | |  | |
|  | |  | |
|  | |  | |
