# Supplementary material for: Network structure underpinning (dys)homeostasis in chronic fatigue syndrome; Preliminary findings
Source: PLoS One. 2019 Mar 25;14(3):e0213724. doi: 10.1371/journal.pone.0213724 (PMC6433252; doi:10.1371/journal.pone.0213724)
Supplement: S1 File — (DOCX) [file pone.0213724.s007.docx]

Supplement 1: Full list of variables

Presented below is a complete list of the variables entered into the original model before stepwise regression was applied:

| Autonomics |  |
| --- | --- |
| Heart rate |  |
| Systolic blood pressure |  |
| Diastolic blood pressure |  |
| Cardiac index | The amount of blood pumped by the heart in one minute, per metre squared of body surface area |
| Total peripheral resistance index | Resistance of blood vessels to left ventricular action per metre squared of body surface area |
| Left ventricular work index | Left ventricular work in each heartbeat per metre squared of body surface area |
| Thoracic fluid content | The total internal and external fluid content of the chest |
| Low frequency heart rate variability | The low frequency power band of decomposed heart rate variability. This is an indicator of baroreflex effectiveness |
| High frequency heart rate variability | The high frequency power band of decomposed heart rate variability. This is an indicator of vagal power |
| Slope mean | The mean of all regression slopes describing change in heart rate over change in blood pressure |
| Baroreflex effectiveness index | The ratio of changes in blood pressure followed by changes in heart rate to the total number of changes in blood pressure |
|  |  |
| Cytokines |  |
| IL-21 | Pro-inflammatory cytokine which regulates the function of T and B cells, natural killer cells and myeloid cells |
| IFN-α | Type 1 interferon which mediates the innate immune response against viral infection. |
| IFN-γ | Mediates macrophage and natural killer cell activation and inhibits the proliferation of T-helper cells type 2. |
| Il-17 | Pro-inflammatory cytokine which also induces the production of neutrophils and chemokines. |
| TNF-α | Pro-inflammatory cytokine involved in the regulation of macrophage activity. |
| LTA | Pro-inflammatory cytokine involved in the development of lymphoid tissue and |
| IL-6 | Cytokine with pro and anti-inflammatory properties depending on its mode of signalling |
| IL-1β | Pro-inflammatory cytokine which activates macrophages and T-lymphocytes. |
| IL-10 | Cytokine which suppresses macrophage and T-cell activity and enhances activity of anti-inflammatory agents. |
|  |  |
| HPA axis measures |  |
| Saliva cortisol at 10:00 (measured on day1 and day2) |  |
| Saliva cortisol at 10:30 (measured on day1 and day2) |  |
| Saliva cortisol at 11:00 (measured on day1 and day2) |  |
| Saliva cortisol at 11:30 (measured on day1 and day2) |  |
| Saliva cortisol at 12:00 (measured on day1 and day2) |  |
| AUCg (day1 and day2) | The total cortisol output (calculated via trapezoid integration) over the testing session |
| AUCi (day1 and day2) | The rate of change of cortisol output over the testing session |
| NULL | The glucocorticoid receptor binding affinity in untreated peripheral blood samples |
| LPS | The glucocorticoid receptor binding affinity in blood samples treated with lipopolysaccharide |
| Dex10 | The glucocorticoid receptor binding affinity in blood samples treated with LPS solution and 10nmol dexamethasone solution |
| Dex100 | The glucocorticoid receptor binding affinity in blood samples treated with LPS solution and 100nmol dexamethasone solution |
